# Xanthogranulomatous Endometritis with calculus formation in setting of prolapsed uterus

**DOI:** 10.4322/acr.2023.439

**Published:** 2023-07-19

**Authors:** Nikhil Kumar, Prima Shuchita Lakra, Ranwir Kumar Sinha, Asitava Deb Roy, Debarshi Saha, Jitendra Kumar Sinha

**Affiliations:** 1 All India Institute of Medical Sciences, Pathology/Lab Medicine, Deoghar, Jharkhand, India; 2 IQ City Medical College and Hospital, Pathology/Lab Medicine, Durgapur, West Bengal, India

**Keywords:** Endometritis, Endometrium, Histiocytes, Uterine Prolapse

## Abstract

Xanthogranulomatous inflammation is a rare benign inflammatory lesion characterized by sheets of lipid-laden foamy histiocytes. It has been reported in various organs, mainly the kidney and gall bladder. Xanthogranulomatous endometritis (XGE) is sporadic, with only a few cases reported in the English medical literature. Herein, we report a case of xanthogranulomatous endometritis with the formation of stones in a 50-year-old female patient with a prolapsed uterus. Grossly the endometrium was irregular, and the uterine cavity was filled with a yellow friable material, a polypoid growth, and yellowish stones. The microscopy showed sheets of histiocytes with few preserved endometrial glands. In this case, the xanthogranulomatous inflammation may mimic a clear cell carcinoma involving the endometrium and myometrium. One of the important differential diagnoses is malakoplakia. Immunohistochemistry and special stains are helpful in diagnosis.

## INTRODUCTION

Xanthogranulomatous inflammation (XGI) is a rare but well-described entity first reported in the genitourinary tract. It is commonly seen in the kidney and gall bladder but can involve any organ.^1^ In the female genital tract, XGI most widely involves the endometrium and the adnexa as endometritis and tubo-ovarian mass, respectively.^2,3^ Xanthogranulomatous endometritis (XGE) is sporadic, with only a few cases reported in the English medical literature, and usually seen in postmenopausal women with endometrial hyperplasia, endometrial cancer and /or cervical stenosis.^4^ XGE occurs mostly in postmenopausal women.^5^ XGE can clinically and radiologically mimic malignancy because of its gross presentation as a friable tissue.^1^ Hence, knowledge of this inflammatory condition is of utmost importance for the pathologist, radiologist, and clinician to avoid a false diagnosis of malignancy which may cause unnecessary anxiety. Herein, we report a unique case of xanthogranulomatous endometritis with stone formation in a 50-year-old female with a prolapsed uterus.

## CASE PRESENTATION

**Figure 1:**
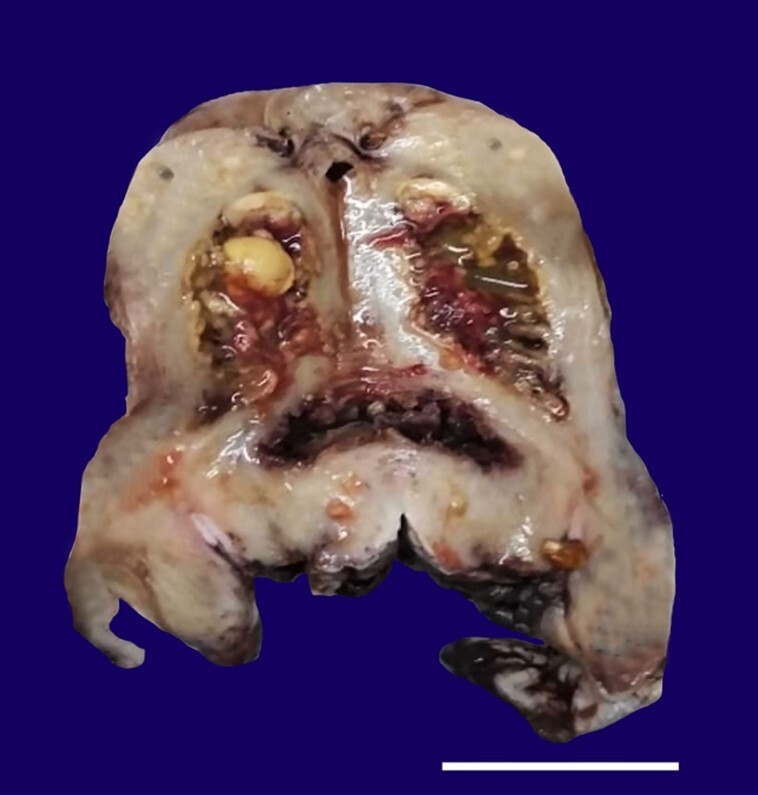
Gross view of the surgical specimen’s cut surface showing the irregular endometrium lined by yellowish friable material. The endometrial cavity shows the presence of yellow-colored stones (scale bar= 4,5 cm).

A 50-year-old postmenopausal woman presented with complaints of recent vaginal bleeding and something coming out of the vagina. There was no previous history of vaginal bleeding and discharge. A gynecological examination revealed prolapsed uterus with an ulcer in the posterior lip of the cervix, which bled on touch. The lower abdomen ultrasonography showed the presence of an ill-defined hyperechoic polypoidal lesion within the uterine cavity. She underwent a vaginal hysterectomy for a prolapsed uterus. On gross examination, the endometrium was irregular, and the cavity was filled with a yellowish friable material, a polypoid growth, and yellow-colored stones (Figure 1). The myometrium was grossly unremarkable.

**Figure 2:**
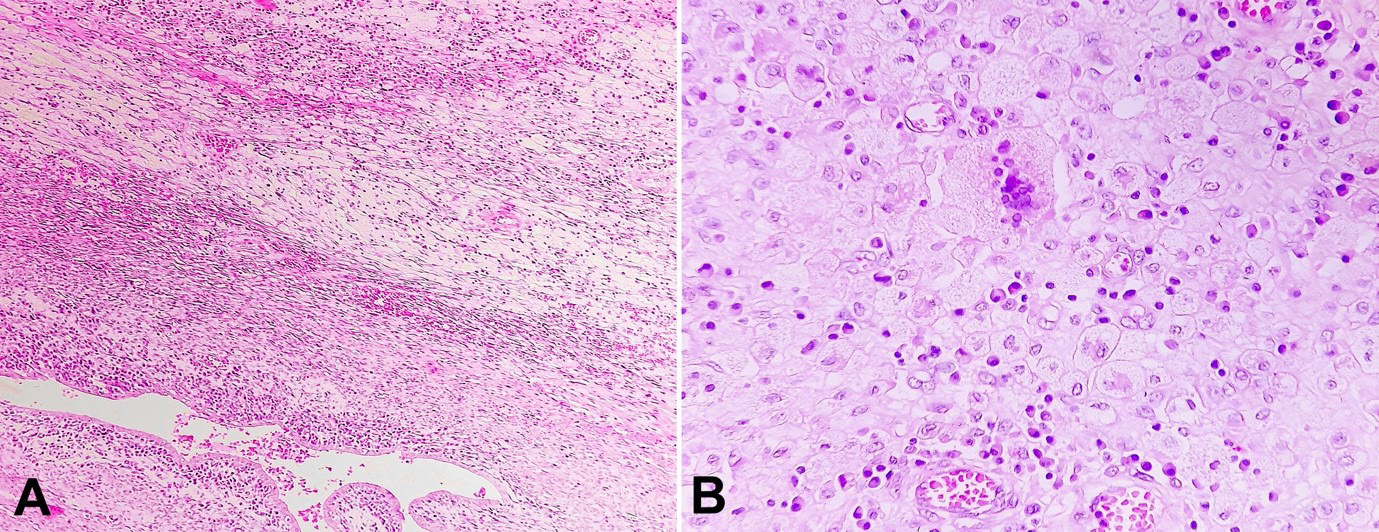
Photomicrographs of the uterus. **A -** uterine wall showing occasional endometrial glands and features of xanthogranulomatous endometritis. (H&E, X 40); **B -** endometrium showing sheets of lipid-laden foamy macrophages and other inflammatory cells (H&E, X 40).

Microscopically, the endometrium showed sheets of lipid-containing histiocytic cells and chronic inflammatory cells. Few well-preserved endometrial glands and multinucleated giant cells were noted, but well-defined granulomas were absent (Figures 2A, and 2B).

The cervix and myometrium were uninvolved. The diagnosis of xanthogranulomatous endometritis (XGE) in this case was made based on the presence of numerous foamy histiocytes together with other chronic inflammatory cells.

## DISCUSSION

XGE is a rarely reported entity characterized by chronic inflammation of the endometrium with the presence of sheets of lipid-laden macrophages. XGE was first reported in the year 1978 by Barua et al.^6^ XGE is also known as histiocytic endometritis.^7,8^

Bleeding, vaginal discharge, and cervical stenosis, sometimes with pyometra, are the most common presenting symptom.^9^ In our case, the patient complained of vaginal bleeding and had a prolapsed uterus. There are cases of XGE reported in literature associated with cervical stenosis and endometrial carcinoma.^10,11^ Grossly, the endometrial lining in cases of XGE is often irregular. In our case, the endometrium was irregular, with a polypoidal growth.^4,6,12^ Ji Min Na et al.^5^ reported two cases of XGE, in which gross examination revealed an irregular yellow endometrial lining like our index case.

The etiology and pathogenesis of XGE are unknown. Russack V et al.^3^ reported a case of XGE in endometrial adenocarcinoma. The cause of the XGE was attributed to tissue necrosis caused by radiotherapy, which might have led to the accumulation of cholesterol and other lipids that were phagocytized by histiocytes resulting in the foam cells formation.

Hussain et al.^13^ suggested that the causes like organ obstruction, suppurative infections, and hemorrhage may lead to xanthogranulomatous inflammation.

Dogan-Ekici I et al.^1^ also suggested the coexistence of XGE with endometrial adenocarcinoma and that the irregular necrotic appearance of the XGE may mimic carcinoma macroscopically. The uterine stone is scarce, with only one case reported by Negussie T and Kidane P^14^ In our case, the stone formation was also seen, and on extensive literature search using the Pubmed search engine and Google Scholar, we found no article on XGE with stone formation.

Xanthogranulomatous inflammation mimics malignancy being misdiagnosed in radiology.^1,15^ Therefore, histopathological examination plays an important role in its diagnosis.

The infiltration of the myometrium by histiocytes can mimic metastatic clear cell carcinoma or myometrial sarcoma. But the cytological characteristics will help to rule out malignancy. Immunohistochemistry is essential in these cases as positive CD68 lipid-laden macrophages and positive CD3 and CD20 lymphocytes rule out malignancy.^10^

Xanthogranulomatous inflammation mainly involves the kidney and gall bladder more frequently and rarely consists of the endometrium, breast, colon, and nasal cavities.^13,16-19^

The differential diagnosis of XGE involves malakoplakia. Malakoplakia commonly affects the genitourinary tract, such as the bladder, prostate, ureter, kidney, female genital tract, and retroperitoneal tissue may be involved.^20^ It is characterized histologically by von Hansemann histiocytes and Michaelis - Gutmann bodies.^21^ In our case, Michaelis-Gutmann bodies were absent in histochemical PAS and Prussian blue staining. Therefore, the diagnosis of malakoplakia was excluded.

Awareness of xanthogranulomatous endometritis is important for pathologists and gynecologists since it can mimic malignancy both radiologically and grossly. It is recommended that endometrium be sampled extensively.

## References

[1] Doğan-Ekici AI, Usubütün A, Küçükali T, Ayhan A (2007). Xanthogranulomatous Endometritis: a challenging imitator of endometrial carcinoma. Infect Dis Obstet Gynecol.

[2] Chou SC, Wang JS, Tseng HH (2002). Malacoplakia of the ovary, fallopian tube, and uterus: a case associated with diabetes mellitus. Pathol Int.

[3] Russack V, Lammers RJ (1990). Xanthogranulomatous endometritis. Report of six cases and a proposed mechanism of development. Arch Pathol Lab Med.

[4] Du XZ, Lu M, Safneck J, Baker P, Dean E, Mottola J (2018). Xanthogranulomatous endometritis mimicking endometrial carcinoma: A case report and review of literature. Radiol Case Rep.

[5] Na JM, Kim MH, Ko GH, Shin JK (2020). Xanthogranulomatous endometritis: a report of two Korean cases with cytologic findings. J Pathol Transl Med.

[6] Barua R, Kirkland JA, Petrucco OM (1978). Xanthogranulomatous endometritis: case report. Pathology.

[7] Moukassa D, Maurage CA, Leroy X, Leory-Billiard M, Farine MO, Janin-Mercier A (1998). Spontaneously resolved female infertility associated with histiocytic endometritis. Ann Pathol.

[8] Buckley CH, Fox H (1980). Histiocytic endometritis. Histopathology.

[9] Zhang X, Dong H, Zhang L, Desouki MM, Zhao C (2012). Xanthogranulomatous Inflammation of the female genital tract: report of three cases. J Cancer.

[10] Merviel P, James P, Carlier M (2021). Xanthogranulomatous endometritis: a case report and literature review. Clin Case Rep.

[11] Pounder DJ, Iyer PV (1985). Xanthogranulomatous endometritis associated with endometrial carcinoma. Arch Pathol Lab Med.

[12] Malik V, Chatterjee D, Goel B, Takkar N. (2019). Xanthogranulomatous endometritis: a benign uncommon masquerader of malignancy. J -Life Health.

[13] Hussain T, Elahi B, Long E, Mahapatra T, McManus PL, Kneeshaw PJ (2012). Xanthogranulomatous inflammation involving latissimus dorsi donor site and implant breast reconstruction: case report and literature review. World J Surg Oncol.

[14] Negussie T, Gebremedhin P (2013). A uterine stone: a case report. East Cent Afr J Surg.

[15] Makkar M, Singh D, Gill M (2013). Xanthogranulomatous endometritis: an unusual pathological entity mimicking endometrial carcinoma. Ann Med Health Sci Res.

[16] Dhawan S, Jain D, Kalhan SK (2011). Xanthogranulomatous inflammation of ascending colon with mucosal involvement: report of a first case. J Crohns Colitis.

[17] Corless K, Samy A, Kamil A, Hogan AM (2020). Benign cyst with xanthogranulomatous inflammation involving the transverse colon and the common iliac artery. J Surg Case Rep.

[18] Chieco PA, Antolino L, Giaccaglia V (2014). Acute abdomen: rare and unusual presentation of right colic xanthogranulomatosis. World J Gastroenterol.

[19] Boon WJ, Vong KS, Tang IP (2022). Bilateral xanthogranulomatous inflammation of nasal and nasopharynx mass: a case report. Egypt J Otolaryngol.

[20] Dong H, Dawes S, Philip J, Chaudhri S, Subramonian K (2014). Malakoplakia of the urogenital tract. Urol Case Rep.

[21] McClure J (1979). Malakoplakia of the prostate: a report of two cases and a review of the literature. J Clin Pathol.

